# Supplementary material for: Interaction between subventricular zone microglia and neural stem cells impacts the neurogenic response in a mouse model of cortical ischemic stroke
Source: Nat Commun. 2024 Oct 24;15:9095. doi: 10.1038/s41467-024-53217-1 (PMC11502905; doi:10.1038/s41467-024-53217-1)
Supplement: Supplementary file 1 — Supplementary Information [file 41467_2024_53217_MOESM1_ESM.pdf]

Interaction between subventricular zone microglia and neural stem cells impacts the neurogenic response in a mouse model of cortical ischemic stroke

Nath *et al.*

Supplementary Information

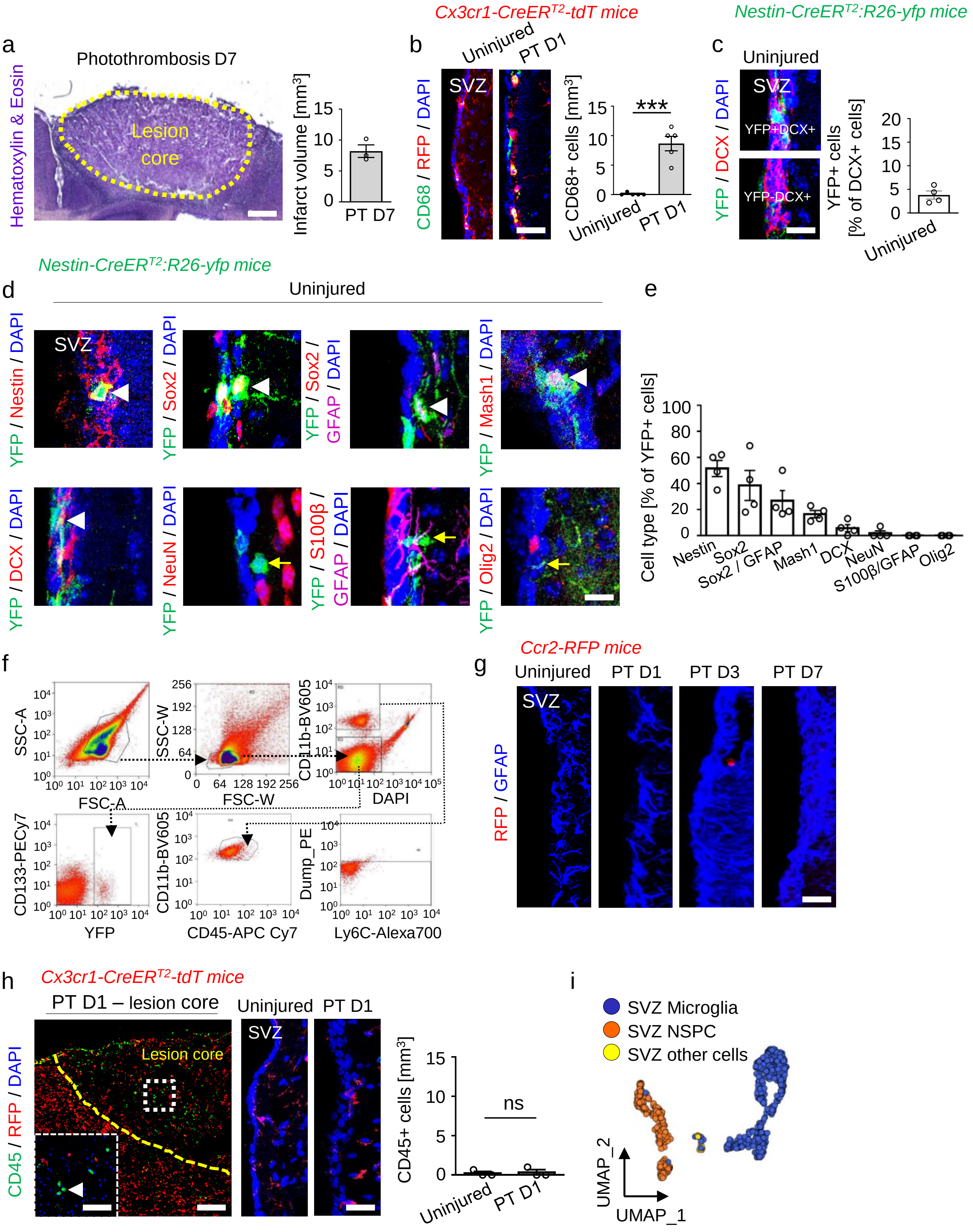
a
Photothrombosis D7
Hematoxylin & Eosin
Lesion core
Infarct volume [mm³]
15
10
5
0
PT D7
b
Cx3cr1-CreERT2-tdT mice
Uninjured
PT D1
SVZ
CD68 / RFP / DAPI
CD68+ cells [mm³]
***
Uninjured
PT D1
c
Nestin-CreERT2:R26-yfp mice
Uninjured
SVZ
YFP+DCX+
YFP-DCX+
YFP / DCX / DAPI
YFP+ cells [% of DCX+ cells]
20
15
10
5
0
Uninjured
d
Nestin-CreERT2:R26-yfp mice
Uninjured
SVZ
YFP / Nestin / DAPI
YFP / Sox2 / DAPI
YFP / Sox2 / GFAP / DAPI
YFP / Mash1 / DAPI
YFP / DCX / DAPI
YFP / NeuN / DAPI
YFP / S100β / GFAP / DAPI
YFP / Olig2 / DAPI
e
Cell type [% of YFP+ cells]
100
80
60
40
20
0
Nestin
Sox2
Sox2 / GFAP
Mash1
DCX
NeuN
S100β/GFAP
Olig2
f
SSC-A
FSC-A
SSC-W
FSC-W
CD11b-BV605
DAPI
CD133-PECy7
YFP
CD11b-BV605
CD45-APC Cy7
Dump_PE
Ly6C-Alexa700
g
Ccr2-RFP mice
Uninjured
PT D1
PT D3
PT D7
SVZ
RFP / GFAP
h
Cx3cr1-CreERT2-tdT mice
PT D1 – lesion core
Lesion core
CD45 / RFP / DAPI
Uninjured
PT D1
SVZ
CD45+ cells [mm³]
ns
Uninjured
PT D1
i
SVZ Microglia
SVZ NSPC
SVZ other cells
UMAP_2
UMAP_1

Supplementary Fig. 1. Cluster identification of the scRNA-Seq dataset. a, Hematoxylin and eosin staining of brain sections 7 days after PT. Dotted yellow line delineates lesion core. Scale, 240 µm. Quantification of the infarct volume of mice at day 7 after PT (n = 3 mice). **b**, Immunolabeling for CD68 and RFP in the SVZ 1 day after PT. Scale, 36 µm. Quantification of the number of CD68+ cells in the SVZ 1 day after PT (n = 5). **c**, Immunolabeling for YFP and DCX in the SVZ of uninjured *Nestin-CreER*^{T2}*:R26-yfp* mice 12 days after the last tamoxifen injection. Scale, 12 µm. Quantification of the percentage of YFP+DCX+ cells relative to all DCX+ cells (n = 4). **d**, Immunolabeling for YFP with Nestin, Sox2, GFAP, Mash1, DCX, NeuN, S100β, and Olig2 labeling in the SVZ of uninjured *Nestin-CreER*^{T2}*:R26-yfp* mice. Arrowheads indicate YFP+marker+ and arrows indicate YFP+marker− cells for the marker combinations. Scale, 8 µm. **e.** Quantification of percentage of cell types per total YFP+ cells in the SVZ (n = 4). **f**, FACS isolation of SVZ YFP+ NSPCs and CD11b+CD45^{low} microglia. **g**, Immunolabeling for RFP+ blood-derived circulating monocytes (red) in the SVZ at the indicated times after PT and in uninjured *Ccr2-RFP* reporter mice (n = 5 mice). Scale, 18 µm. **h**, Immunolabeling for CD45 and RFP in the lesion core (left) and in the SVZ 1 day after PT. Dashed box indicates magnified area showing CD45+ cells in the lesion core (arrowhead). Scales, 180 µm (lesion core), left; 90 µm, magnification; 30 µm, SVZ. Quantification of CD45+ cells in the SVZ in uninjured mice and 1 day after PT (n = 3 mice). **i**, UMAP representation of 658 individual NSPCs and microglia from the SVZ 1 and 7 days after PT and uninjured mice measured by scRNA-Seq. Each dot represents an individual cell. All plots show the mean ± SEM. ns, no significant; ***P<0.001, unpaired Student's *t*-tests (b, h). SVZ, subventricular zone. Source data are provided as a Source Data file.

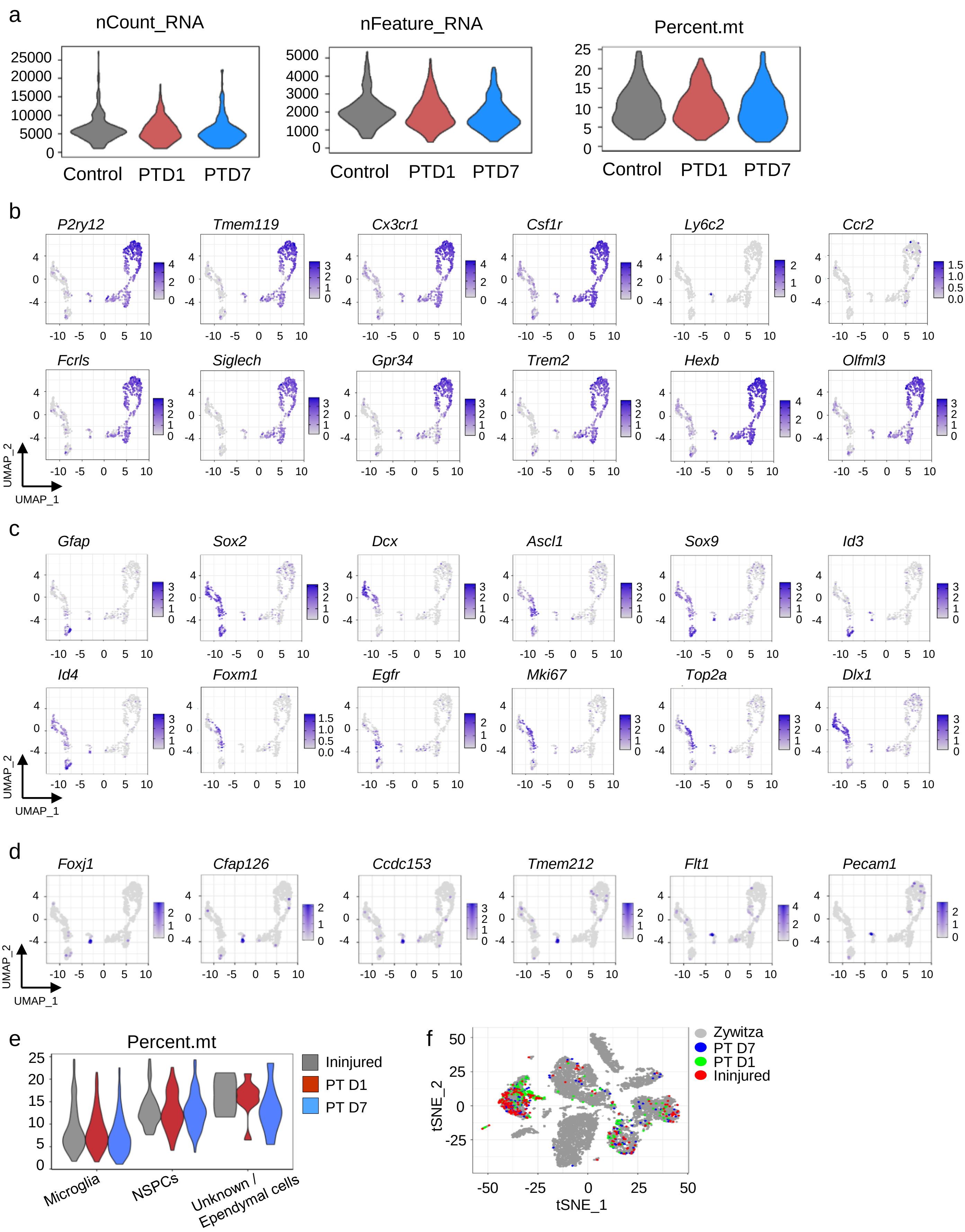

a
nCount_RNA
nFeature_RNA
Percent.mt
Control
PTD1
PTD7
b
P2ry12
Tmem119
Cx3cr1
Csf1r
Ly6c2
Ccr2
Fcrls
Siglech
Gpr34
Trem2
Hexb
Olfml3
UMAP_2
UMAP_1
c
Gfap
Sox2
Dcx
Ascl1
Sox9
Id3
Id4
Foxm1
Egfr
Mki67
Top2a
Dlx1
d
Foxj1
Cfap126
Ccdc153
Tmem212
Flt1
Pecam1
e
Percent.mt
Ininjured
PT D1
PT D7
Microglia
NSPCs
Unknown / Ependymal cells
f
Zywitza
PT D7
PT D1
Ininjured
tSNE_1
tSNE_2

Supplementary Fig. 2. Quality-control metrics and cluster identification for the scRNA-Seq dataset. a, Violin plots depicting number of genes and unique features related to genes, and percentage of transcripts mapping to the mitochondrial genome 1 and 7 days after PT and in the uninjured control. **b**, UMAP plots colored for expression of canonical genes to verify the microglial cluster. **c**, UMAP plots colored for expression of canonical genes to verify the NSPC cluster. **d**, UMAP plots colored for expression of canonical genes to verify the mixed cell population cluster. **e**, Violin plots of mitochondrial genes enriched in different cell populations. **f**, t-SNE plot of microglia cells with the time point of origin, compared with Zywitza's study[11].

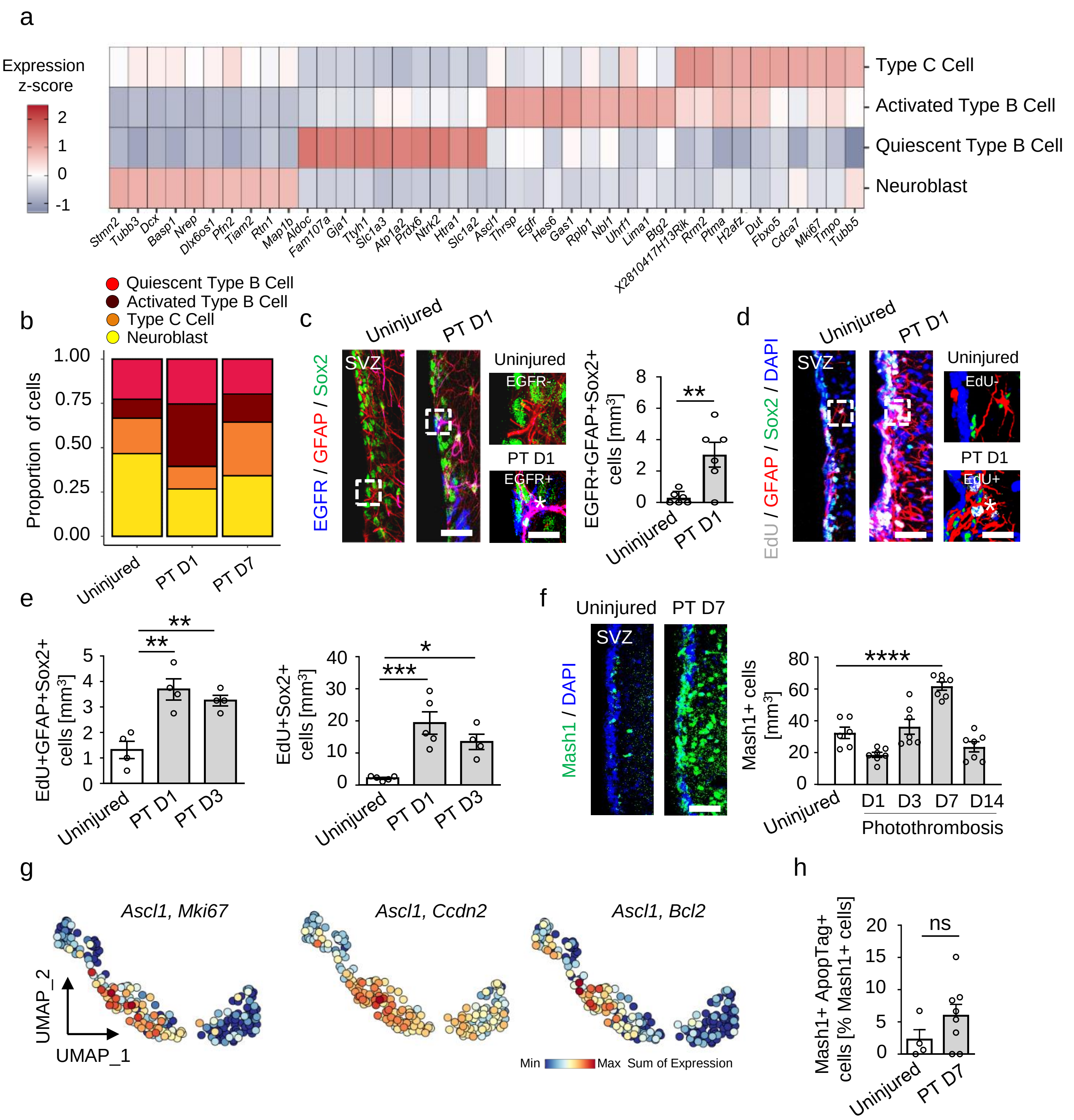

a
Expression z-score
2
1
0
-1
Type C Cell
Activated Type B Cell
Quiescent Type B Cell
Neuroblast
Stmn2 Tubb3 Dcx Basp1 Nrep Dlx6os1 Pfn2 Tiam2 Rtn1 Map1b Aldoc Fam107a Gja1 Ttyh1 Slc1a3 Atp1a2 Prdx6 Ntrk2 Htra1 Slc1a2 Ascl1 Thrsp Egfr Hes6 Gas1 Rplp1 Nbl1 Uhrf1 Lima1 Btg2 X2810417H13Rik Rrm2 Ptma H2afz Dut Fbxo5 Cdca7 Mki67 Tmpo Tubb5
b
Quiescent Type B Cell
Activated Type B Cell
Type C Cell
Neuroblast
Proportion of cells
1.00
0.75
0.50
0.25
0.00
Uninjured
PT D1
PT D7
c
Uninjured
PT D1
EGFR / GFAP / Sox2
SVZ
Uninjured
EGFR-
PT D1
EGFR+
EGFR+GFAP+Sox2+ cells [mm³]
8
6
4
2
0
**
Uninjured
PT D1
d
Uninjured
PT D1
EdU / GFAP / Sox2 / DAPI
SVZ
Uninjured
EdU-
PT D1
EdU+
e
EdU+GFAP+Sox2+ cells [mm³]
5
4
3
2
1
0
**
**
Uninjured
PT D1
PT D3
EdU+Sox2+ cells [mm³]
40
30
20
10
0
*
***
Uninjured
PT D1
PT D3
f
Uninjured
PT D7
SVZ
Mash1 / DAPI
Mash1+ cells [mm³]
80
60
40
20
0
****
Uninjured
D1
D3
D7
D14
Photothrombosis
g
Ascl1, Mki67
Ascl1, Ccdn2
Ascl1, Bcl2
UMAP_2
UMAP_1
Min
Max
Sum of Expression
h
Mash1+ ApopTag+ cells [% Mash1+ cells]
20
15
10
5
0
ns
Uninjured
PT D7

Supplementary Fig. 3. NSPC activation and apoptosis in the SVZ after cortical stroke. **a**, Heat map of the top-10 differentially expressed genes (DEGs) in NSPC clusters. **b**, Quantification of NSPC cluster proportions 1 and 7 days after PT and in uninjured mice. **c**, Immunolabeling for EGFR, GFAP, and Sox2 in uninjured mice and 1 day after PT. The white dashed boxes indicate magnification of EGFR−GFAP+Sox2+ quiescent type B (right, top) and EGFR+GFAP+Sox2+ activated type B cells (star) (right, bottom) in the SVZ in an uninjured mouse and 1 day after PT, respectively. Scales, 25 μm, left; 12 μm, magnification. Quantification of EGFR+GFAP+Sox2+ cells in the SVZ per area 1 day after PT and in uninjured mice (n = 6). **d**, Immunolabeling for EdU, GFAP, and Sox2 in the SVZ in uninjured mice and 1 day after PT. Dashed boxes indicate the magnification of an EdU−GFAP+Sox2+ quiescent type B cell (right, top) and an EdU+GFAP+Sox2+ activated type B cell (star) (right, bottom) in uninjured mice and 1 day after PT, respectively. Scale bars, 27 μm, left; 10 μm, magnification. **e**, Quantification of EdU+GFAP+Sox2+ (n = 4) and EdU+Sox2+ (n = 5 mice, uninjured, PT D1; n = 4, PT D3) cells in the SVZ per area 1 day and 3 days after PT and in uninjured mice. **f**, Immunolabeling for Mash1 (green) in the SVZ 7 days after PT, compared with uninjured mice. Scale, 27 μm. Quantification of Mash1+ cells in the SVZ per area 1, 3, 7, and 14 days after PT, and in uninjured mice (n = 6, uninjured; n = 7, PT D1-D14). **g**, *Ascl1*+ proliferative type C cells co-express *Mki67*, *Ccdn2*, and *Bcl2*, as in the projected expression of the UMAP after PT. **h**, Quantification of Mash1+ApopTag+ cell percentage in the SVZ 7 days after PT, compared with uninjured mice (n = 4, uninjured; n = 8, PT D7). All graphs show the mean ± SEM. *P<0.05, **P<0.01, ***P<0.01, ****P<0.0001, ns not significant, one-way ANOVAs with Bonferroni corrections for multiple comparisons (e, f) and unpaired Student's *t*-tests (c, h). SVZ, subventricular zone. Source data are provided as a Source Data file.

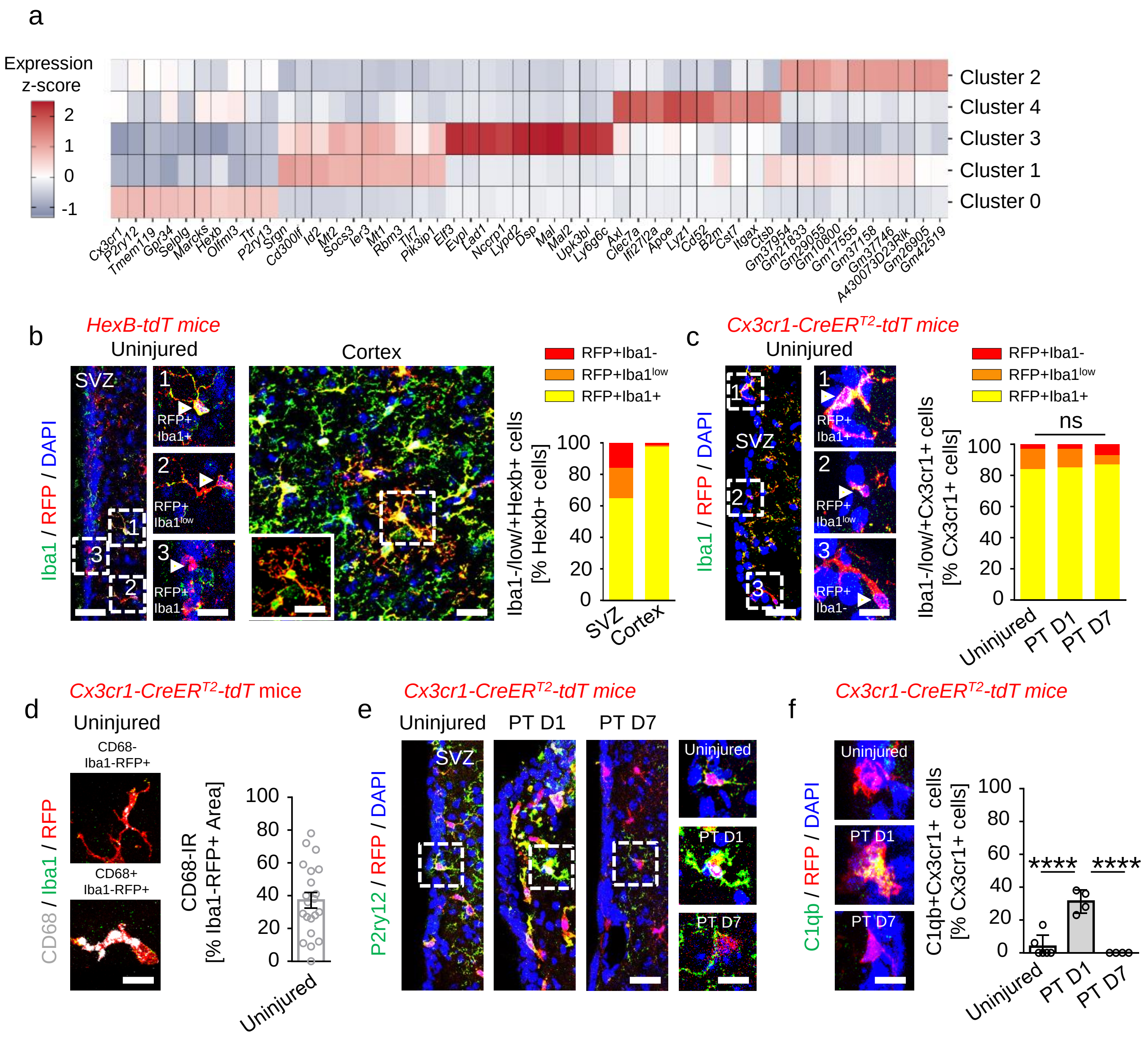

a
Expression z-score
2
1
0
-1
Cluster 2
Cluster 4
Cluster 3
Cluster 1
Cluster 0
Cx3cr1
P2ry12
Tmem119
Gpr34
Selplg
Marcks
Hexb
Olfml3
Ttr
P2ry13
Srgn
Cd300lf
Id2
Mt2
Socs3
Ier3
Mt1
Rbm3
Tlr7
Pik3ip1
Elf3
Evpl
Lad1
Nccrp1
Lypd2
Dsp
Mal
Mal2
Upk3bl
Ly6g6c
Axl
Clec7a
Ifi27l2a
Apoe
Lyz1
Cd52
B2m
Cst7
Itgax
Ctsb
Gm37954
Gm21833
Gm29055
Gm10800
Gm17555
Gm37158
Gm37746
A430073D23Rik
Gm26905
Gm42519
b
HexB-tdT mice
Uninjured
Cortex
SVZ
Iba1 / RFP / DAPI
RFP+ Iba1+
RFP+ Iba1low
RFP+ Iba1-
RFP+Iba1-
RFP+Iba1low
RFP+Iba1+
Iba1-/low/+Hexb+ cells [% Hexb+ cells]
SVZ
Cortex
c
Cx3cr1-CreERT2-tdT mice
Uninjured
ns
Iba1-/low/+Cx3cr1+ cells [% Cx3cr1+ cells]
Uninjured
PT D1
PT D7
d
Cx3cr1-CreERT2-tdT mice
Uninjured
CD68- Iba1-RFP+
CD68+ Iba1-RFP+
CD68 / Iba1 / RFP
CD68-IR [% Iba1-RFP+ Area]
e
Cx3cr1-CreERT2-tdT mice
P2ry12 / RFP / DAPI
f
Cx3cr1-CreERT2-tdT mice
C1qb / RFP / DAPI
C1qb+Cx3cr1+ cells [% Cx3cr1+ cells]
****
****

Supplementary Fig. 4. SVZ microglial molecular heterogeneity after PT. a, Heat map of the top-10 DEGs in microglial clusters. **b**, Immunolabeling for Iba1 and RFP in the SVZ (left) and cortex (right) of uninjured *HexB-tdTomato* mice. White boxes indicate magnifications of a RFP+Iba1+ cell (left, top), a RFP+Iba1^{low} cell (left, middle) and a RFP+Iba1− cell (left, bottom) in the SVZ, and a RFP+Iba1+ cell (right, bottom) in the cortex. Examples of each cell type are indicated by arrowheads. Scales, 20 µm, SVZ; 10 µm, magnifications of SVZ cells; 35 µm, cortex; 8 µm, magnification of the cortical cell. Quantification of the percentage of RFP+Iba1+, RFP+Iba1^{low} and RFP+Iba1− cells in the SVZ and cortex of uninjured mice (n = 4 mice). **c**, Immunolabeling for Iba1 and RFP in the SVZ of uninjured *Cx3cr1-CreER^{T2}:R26-tdTomato* mice. Boxes indicate magnifications of RFP+Iba1+ (right, top), RFP+Iba1^{low} cells (right, middle) and a RFP+Iba1− cell (right, bottom) in the SVZ. Examples of each type of cell are indicated by the white arrowheads. Scales, 33 µm, SVZ; 9 µm, magnifications. Quantification of the percentage of RFP+Iba1+, RFP+Iba1^{low}, and RFP+Iba1− cells in the SVZ of uninjured mice and mice 1 day and 7 days after PT (n = 3). **d**, Immunolabeling for CD68, Iba1, and RFP in the SVZ of uninjured *Cx3cr1-CreER^{T2}:R26-tdTomato* mice representing CD68+Iba1−RFP+ (bottom) and CD68−Iba1−RFP+ cells (top). Scale, 12 µm. Quantification of the CD68 immunoreactivity (IR) in Iba1-RFP+ cells in the SVZ of uninjured mice (n = 3 mice, a total of 21 cells were analyzed). **e**, Immunolabeling for P2ry12 and RFP in the SVZ of uninjured *Cx3cr1-CreER^{T2}:R26-tdTomato* mice and mice 1 and 7 days after PT. Boxes indicate magnification of representative cells showing P2ry12 expression at 1 and 7 days after PT and in uninjured mice (n = 3). Scales, 25 µm; 8 µm, magnifications. **f**, Immunolabeling for C1qb and RFP of uninjured *Cx3cr1-CreER^{T2}:R26-tdTomato* mice and mice 1 and 7 days after PT. Scale, 8 µm. Quantification of the percentage of C1qb^{+} cells of uninjured mice and mice 1 and 7 days after PT (n = 6, uninjured; n = 4, PT D1, PT D7). All graphs show the mean ± SEM. ****P<0.0001, ns not significant, one-way ANOVAs with Bonferroni corrections for multiple comparisons. SVZ, subventricular zone. Source data are provided as a Source Data file.

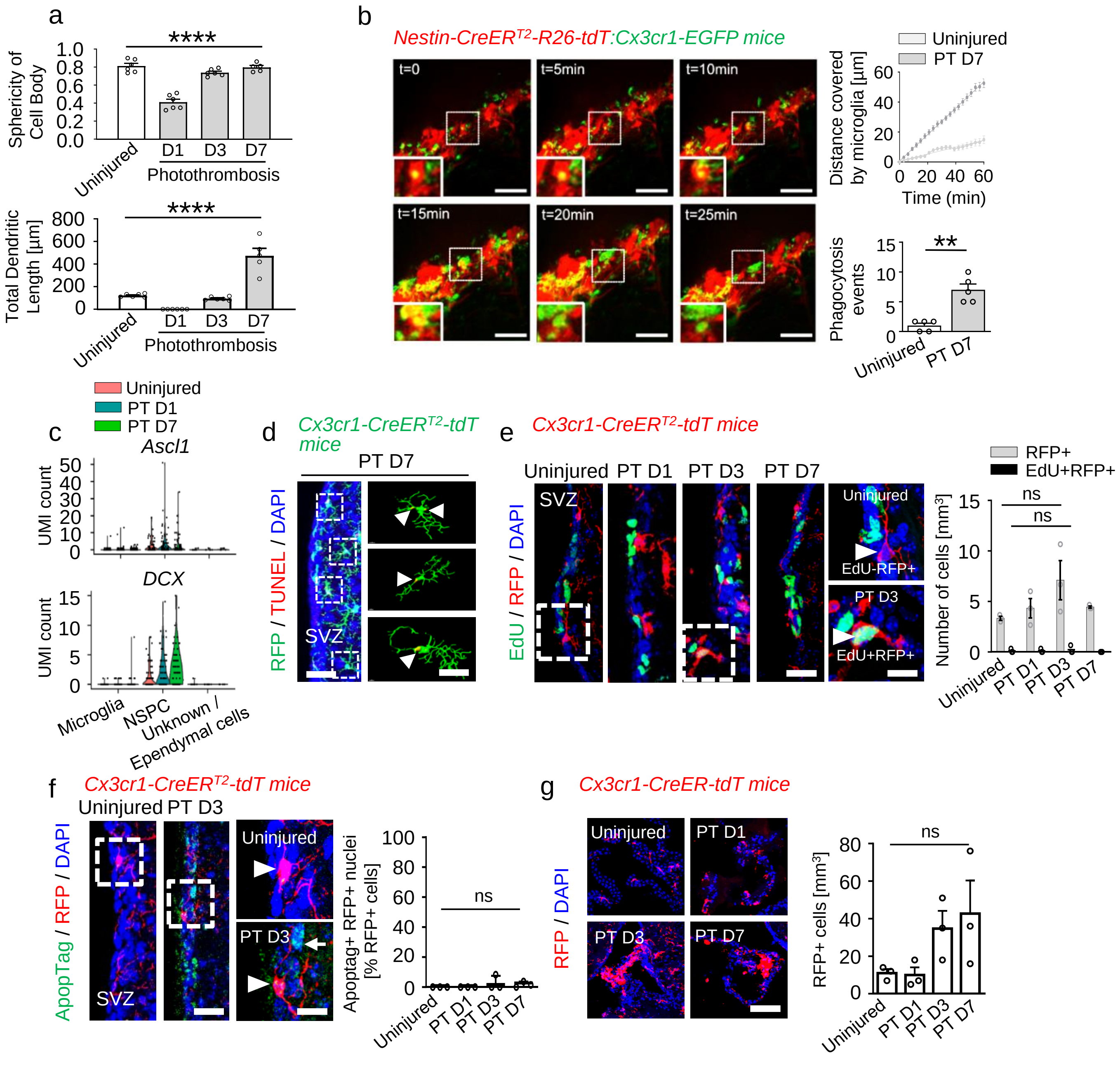

a
Sphericity of Cell Body
1.0 0.8 0.6 0.4 0.2 0.0
****
Uninjured D1 D3 D7
Photothrombosis
Total Dendritic Length [µm]
800 600 400 200 0
****
Uninjured D1 D3 D7
Photothrombosis
b
Nestin-CreER^T2-R26-tdT:Cx3cr1-EGFP mice
t=0 t=5min t=10min t=15min t=20min t=25min
Uninjured
PT D7
Distance covered by microglia [µm]
60 40 20 0
0 20 40 60
Time (min)
Phagocytosis events
15 10 5 0
**
Uninjured PT D7
c
Uninjured
PT D1
PT D7
Ascl1
UMI count
50 40 30 20 10 0
DCX
UMI count
15 10 5 0
Microglia NSPC Unknown / Ependymal cells
d
Cx3cr1-CreER^T2-tdT mice
PT D7
RFP / TUNEL / DAPI
SVZ
e
Cx3cr1-CreER^T2-tdT mice
Uninjured PT D1 PT D3 PT D7
EdU / RFP / DAPI
SVZ
Uninjured
EdU-RFP+
PT D3
EdU+RFP+
RFP+
EdU+RFP+
Number of cells [mm^3]
15 10 5 0
ns
ns
Uninjured PT D1 PT D3 PT D7
f
Cx3cr1-CreER^T2-tdT mice
Uninjured PT D3
ApopTag / RFP / DAPI
SVZ
Uninjured
PT D3
Apoptag+ RFP+ nuclei [% RFP+ cells]
100 80 60 40 20 0
ns
Uninjured PT D1 PT D3 PT D7
g
Cx3cr1-CreER-tdT mice
Uninjured PT D1 PT D3 PT D7
RFP / DAPI
RFP+ cells [mm^3]
80 60 40 20 0
ns
Uninjured PT D1 PT D3 PT D7

Supplementary Fig. 5. Activated SVZ microglia phagocytose dying neuroblasts after PT. a, IMARIS-based automatic quantification of cell morphology of GFP+ microglia 1, 3, and 7 days after PT (n = 6, uninjured, PT D1, PT D3; n = 5, PT D7). **b**, Time-lapse imaging montages revealed phagocytosis of tdT+ NSPCs by GFP+ microglia in the SVZ of *Nestin-CreER^{T2}-R26-tdTomato:Cx3cr1-EGFP* mice. Magnification of regions indicated shows phagocytosis of an NSPC by a microglial cell (bottom insets). Scale, 10 µm. Quantification of velocity dynamics of microglia and phagocytosis events 7 days after PT compared with uninjured control in SVZ acute slices (velocity: n = 2 (35 cells), uninjured; n = 3 (62 cells), 7 days after PT; phagocytosis events: n = 2, uninjured (5 slices); n = 3, 7 days after PT (5 slices). **c**, Analysis of NSPC RNA in microglia. Violin plots for the type C cell and neuroblast genes *Ascl1* and *Dcx* in SVZ microglia. **d**, Immunolabeling for RFP and TUNEL dying cells 7 days after PT. Dashed boxes indicate 3-D IMARIS reconstruction of the spatial localization of TUNEL+ dying cell fragments revealing microglia phagocytosis of dying cells (right, arrowheads) (n = 3). Scales, 30 µm, left; 5 µm, magnifications. **e**, Immunolabeling for EdU and RFP in the SVZ 1, 3, and 7 days after PT and uninjured mice. Dashed boxes indicate magnification of an EdU−RFP+ microglial cell (right, top, arrowhead) and an EdU+RFP+ proliferating microglial cell (right, bottom, arrowhead) in the SVZ in uninjured mice and 3 days after PT, respectively. Scales, 48 µm, left; 14 µm, magnifications. Quantification of RFP+ and EdU+RFP+ cells in the SVZ 1, 3, and 7 days after PT compared to uninjured mice (n = 3). **f**, Immunolabeling for ApopTag and RFP in the SVZ 3 days after PT and uninjured mice. Dashed boxes indicate magnification of ApopTag−RFP+ microglia (arrowheads). An ApopTag+ cell is indicated by an arrow. Scales, 24 µm, left; 12 µm, magnifications. Quantification of the percentage of ApopTag+RFP+ cells in the SVZ 1, 3, and 7 days after PT, compared with uninjured mice (n = 3 mice). **g**, Immunolabeling for RFP in the choroid plexus 1, 3, and 7 days after PT, compared with uninjured mice. Scale, 80 µm. Quantification of RFP+ cells in the choroid plexus in uninjured mice and at different times after PT (n = 3). All graphs show the mean ± SEM. **P<0.01, ****P<0.0001, ns, not significant, one-way ANOVAs with Bonferroni corrections for multiple comparisons (a, e-g), Mann-Whitney test (b). SVZ, subventricular zone. Source data are provided as a Source Data file.

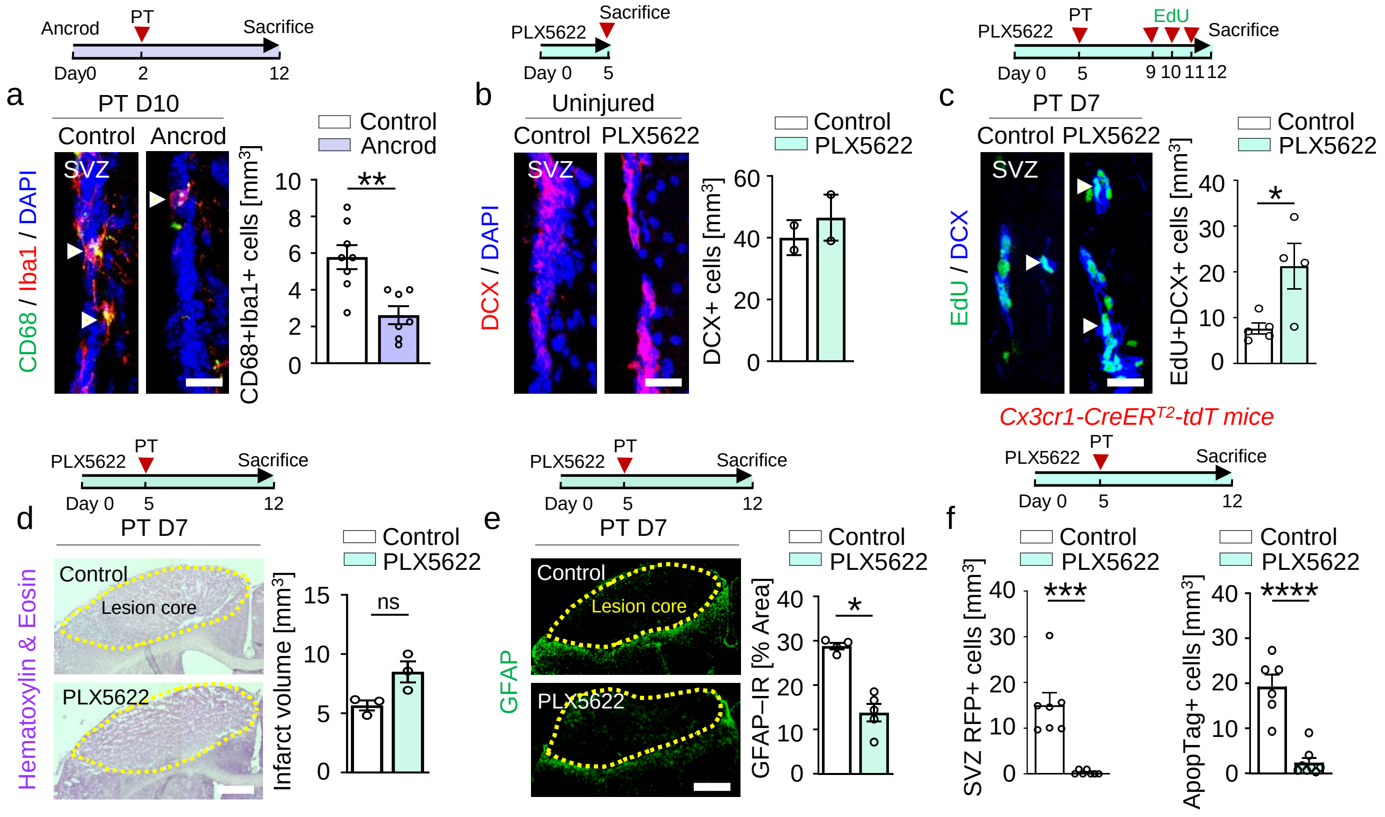

Supplementary Fig. 6. Depletion of SVZ microglia increases the newborn neuroblast cell number after PT. a, Immunolabeling for CD68 and Iba1 in the SVZ of fibrinogen-depleted mice and control mice 10 days after PT. Arrowheads indicate CD68+Iba1+ cells. Scale, 16 μm. Quantification of CD68+Iba1+ cells in the SVZ of fibrinogen-depleted mice and control mice 10 days after PT (n = 8, control; n = 7, ancrod). **b**, Immunolabeling for DCX (red) in the SVZ of PLX5622-fed or control uninjured mice. Scale, 13 μm. Quantification of DCX+ cells in the SVZ in PLX5622-fed or control uninjured mice (n = 2). **c**, Immunolabeling for EdU and DCX in the SVZ of PLX5622-fed or control mice 7 days after PT. Arrowheads indicate EdU+DCX+ cells. Scale bar, 19 μm. Quantification of EdU+DCX+ cells in the SVZ in PLX5622-fed and control mice 7 days after PT (n = 5 mice, control; n = 4 mice, PLX5622). **d**, Hematoxylin and eosin staining of the brain cortex 7 days after PT in PLX5622-treated and control mice. Area delineated by dotted line corresponds to lesion core. Scale, 500 μm. Quantification of infarct volume (n = 3). **e**, Immunolabeling for GFAP in the cortex 7 days after PT in PLX5622-treated and control mice. Area delineated by the dotted yellow line corresponds to the lesion core. Scale bar, 600 μm. Quantification of GFAP-immunoreactivity in the glial border (n = 4, control; n = 5, PLX5622). **f**, Quantification of RFP+ microglia and ApopTag+ cells in the SVZ in PLX5622-fed mice 7 days after PT, compared with control group 7 days after PT (n = 7 mice for microglial quantification (left graph); n = 6, control; n = 8, PLX5622 for ApopTag quantification (right graph)). All graphs show the mean ± SEM. *P<0.05, **P<0.01, ***P<0.001, ****P<0.0001, ns, not significant, Mann-Whitney test (d-e), and unpaired Student's *t*-tests (a, c, f). SVZ, subventricular zone. Source data are provided as a Source Data file.

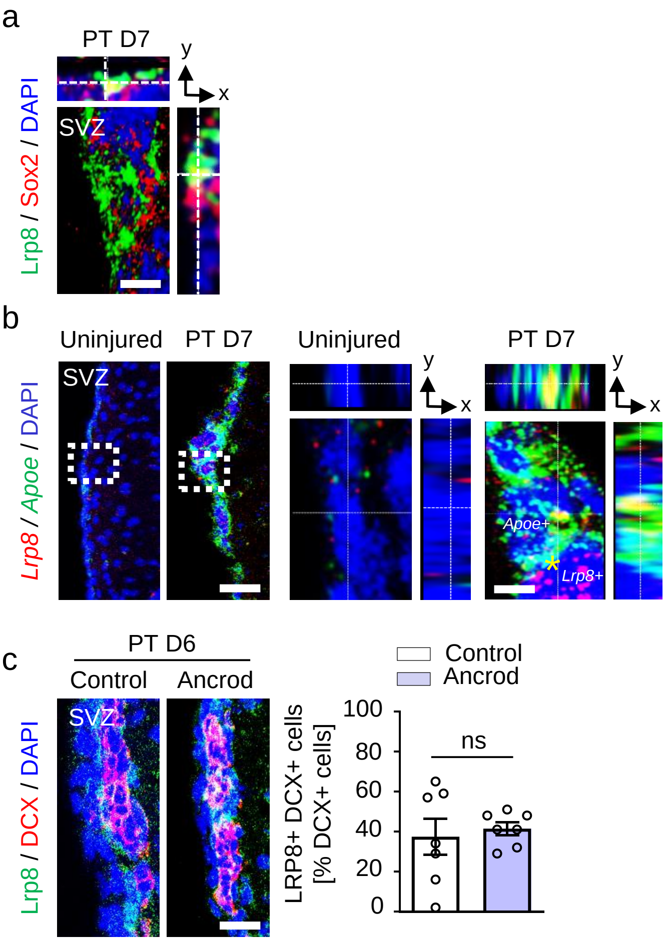

Supplementary Fig. 7. Lrp8 and ApoE expression in SVZ NSPCs and microglia after PT. a, Representative immunolabeling image showing Lrp8 (green) and Sox2 (red) in the SVZ 7 days after PT (n = 4 mice). Scale bar, 10 µm. **b**, *In situ* hybridization for *Lrp8* (red) and *Apoe* (green) 7 days after PT, compared with uninjured mice. White dashed boxes indicate the magnification of an *Lrp8*+ cell next to a *Apoe*+ cell (yellow asterisk) in the SVZ 7 days after PT, compared with *Lrp8*− and *Apoe*− cells in the SVZ of uninjured mice (n = 2 mice, uninjured; n = 4 mice, PT D7). Scale bars, 27 µm, left; 7 µm, right. **c**, Immunolabeling for Lrp8 (green) and DCX (red) in the SVZ in fibrinogen-depleted mice compared with control-treated mice 6 days after PT. Scale bar, 20 µm. Quantification of the percentage of Lrp8+DCX+ cells in fibrinogen-depleted mice compared with control-treated mice 6 days after PT (n = 7 mice). The plot shows the mean ± SEM. ns = not significant, unpaired Student's *t*-tests. SVZ, subventricular zone. Source data are provided as a Source Data file.
