# Supplementary material for: Interaction between subventricular zone microglia and neural stem cells impacts the neurogenic response in a mouse model of cortical ischemic stroke
Source: Nat Commun. 2024 Oct 24;15:9095. doi: 10.1038/s41467-024-53217-1 (PMC11502905; doi:10.1038/s41467-024-53217-1)
Supplement: Supplementary file 3 — Description of Additional Supplementary Files In the "Description of Additional Supplementary Files" please change "Supplementary Table 1:" to "Supplementary Data 1:" [file 41467_2024_53217_MOESM3_ESM.docx]

**Description of Additional Supplementary Files**

**Supplementary Data 1:** Differentially expressed genes (DEGs) of SVZ NSPCs (sheet 1) and microglia (sheet 2), as well as the microglia DEG shared with independent transcriptomic studies of neurodegenerative disease– associated microglia (DAM and IRM, 1 and 7 days after PT (sheet 3) and the full ligand–receptor pair list (sheet 4).

**Supplementary Movie 1:** Representative 3-D view of a GFP+ microglia phagocytosing a tdT+ NSPC in the SVZ 7 days after PT in an acute brain slice from a Nestin-CreERT2- R26-tdTomato:Cx3cr1-EGFP transgenic mouse. Scale bar, 20 μm
