# Supplementary material for: Interaction between subventricular zone microglia and neural stem cells impacts the neurogenic response in a mouse model of cortical ischemic stroke
Source: Nat Commun. 2024 Oct 24;15:9095. doi: 10.1038/s41467-024-53217-1 (PMC11502905; doi:10.1038/s41467-024-53217-1)
Supplement: Supplementary file 6 — Reporting Summary [file 41467_2024_53217_MOESM6_ESM.pdf]

nature portfolio

Corresponding author(s): Schachtrup, Christian

Last updated by author(s): Sep 5, 2024

Reporting Summary

Nature Portfolio wishes to improve the reproducibility of the work that we publish. This form provides structure for consistency and transparency in reporting. For further information on Nature Portfolio policies, see our Editorial Policies and the Editorial Policy Checklist.

Statistics

For all statistical analyses, confirm that the following items are present in the figure legend, table legend, main text, or Methods section.

n/a	Confirmed	
☐	☒	The exact sample size (*n*) for each experimental group/condition, given as a discrete number and unit of measurement
☐	☒	A statement on whether measurements were taken from distinct samples or whether the same sample was measured repeatedly
☐	☒	The statistical test(s) used AND whether they are one- or two-sided<br>*Only common tests should be described solely by name; describe more complex techniques in the Methods section.*
☒	☐	A description of all covariates tested
☐	☒	A description of any assumptions or corrections, such as tests of normality and adjustment for multiple comparisons
☐	☒	A full description of the statistical parameters including central tendency (e.g. means) or other basic estimates (e.g. regression coefficient) AND variation (e.g. standard deviation) or associated estimates of uncertainty (e.g. confidence intervals)
☐	☒	For null hypothesis testing, the test statistic (e.g. *F*, *t*, *r*) with confidence intervals, effect sizes, degrees of freedom and *P* value noted<br>*Give P values as exact values whenever suitable.*
☒	☐	For Bayesian analysis, information on the choice of priors and Markov chain Monte Carlo settings
☒	☐	For hierarchical and complex designs, identification of the appropriate level for tests and full reporting of outcomes
☒	☐	Estimates of effect sizes (e.g. Cohen's *d*, Pearson's *r*), indicating how they were calculated

Our web collection on statistics for biologists contains articles on many of the points above.

Software and code

Policy information about availability of computer code

Data collection	LAS AF image analysis software (Leica), Zen Blue (Zeiss), Gallios FACS (Beckman Coulter), MoFlo XDP cell sorter and the Gallios/Navios analysers (Beckman Coulter), HiSeq3000 Illumina sequencing platform.
Data analysis	Image J (NIH), GraphPad Prism 7, IMARIS (BitPlane), R, R Studio, Seurat R Package (v4.0.2), Single R (v1.6.1), Slingshot R Package 2018, Eulerr R Pacakge, CellPhoneDB, BiomaRt R(v2.48.0), gProfiler (http://biit.cs.ut.ee/gprofiler/gost), ggplot2 R

For manuscripts utilizing custom algorithms or software that are central to the research but not yet described in published literature, software must be made available to editors and reviewers. We strongly encourage code deposition in a community repository (e.g. GitHub). See the Nature Portfolio guidelines for submitting code & software for further information.

Data

Policy information about availability of data

All manuscripts must include a data availability statement. This statement should provide the following information, where applicable:

- Accession codes, unique identifiers, or web links for publicly available datasets
- A description of any restrictions on data availability
- For clinical datasets or third party data, please ensure that the statement adheres to our policy

The scRNA-Seq data generated in this study have been deposited in the NCBI Gene Expression Omnibus database under accession code GSE275939 [https://www.ncbi.nlm.nih.gov/geo/query/acc.cgi?acc=GSE275939]. Source data are provided with this paper. The code used to analyze the data in this study have been deposited under Github [https://github.com/SchachtrupLab/Neuromac_B1.git].

Research involving human participants, their data, or biological material

Policy information about studies with human participants or human data. See also policy information about sex, gender (identity/presentation), and sexual orientation and race, ethnicity and racism.

Reporting on sex and gender	NA
Reporting on race, ethnicity, or other socially relevant groupings	NA
Population characteristics	NA
Recruitment	NA
Ethics oversight	NA

Note that full information on the approval of the study protocol must also be provided in the manuscript.

Field-specific reporting

Please select the one below that is the best fit for your research. If you are not sure, read the appropriate sections before making your selection.

[x] Life sciences [ ] Behavioural & social sciences [ ] Ecological, evolutionary & environmental sciences

For a reference copy of the document with all sections, see nature.com/documents/nr-reporting-summary-flat.pdf

Life sciences study design

All studies must disclose on these points even when the disclosure is negative.

Sample size	Sample size calculations are based on previously published data as stated for the different experiments in the Methods or Results section. No statistical methods were used to predetermine sample size.
Data exclusions	All data points and animal were included in the analysis.
Replication	All number of replication of the experiments are detailed in the figure legends. Sample size calculations are based in previously published data as stated for the different experiments in the methods or results section. All attempts of data replications were successful. Histology was replicated by independent researchers. Data represent biological replicates as indicated in the Figure legends.
Randomization	Wherever applicable, animals were randomly assigned to the different experimental groups.
Blinding	Investigators were not blinded to group allocation during data collection. For scRNA-seq analysis, blinding was not applicable as all measurements were automated such that bias would not be introduced. All in vivo experiments were not blinded because the experimental conditions were evident from image data.

Reporting for specific materials, systems and methods

We require information from authors about some types of materials, experimental systems and methods used in many studies. Here, indicate whether each material, system or method listed is relevant to your study. If you are not sure if a list item applies to your research, read the appropriate section before selecting a response.

Materials & experimental systems

n/a	Involved in the study	
	x	Antibodies
x		Eukaryotic cell lines
x		Palaeontology and archaeology
	x	Animals and other organisms
x		Clinical data
x		Dual use research of concern
x		Plants

Methods

n/a	Involved in the study	
x		ChIP-seq
	x	Flow cytometry
x		MRI-based neuroimaging

Antibodies

Antibodies used

For IHC: anti-Iba1 (1:500, Wako, 019-19741), goat anti-CD31 (1:500, R&D Systems, AF3628-SP), guinea pig anti-Doublecortin (1:1000, Millipore, AB2253), rat anti-GFAP (1:2000, Invitrogen, 13-0300), goat anti-GFP (1:2000, Abcam, ab5450), rabbit anti-GFP (1:2000, Abcam, ab290), sheep anti-Fibrinogen (1:500, US Biological, F4203-02F), goat anti-Nestin (1:200, Santa Cruz, sc-21249), goat anti-Nestin (1:500, Antibodies-Online, ABIN188165), rabbit anti-TMEM119 (1:500, Abcam, ab209064), rat anti-CD68 (1.200, Biorad, MCA1957), rabbit anti-C1qb (1:200, Abcam, ab182451), rabbit anti-P2ry12 (1:500, Sigma Life Science, HPA014518), rabbit anti-Sox2 (1.2000, Abcam, ab97959), rabbit anti-Mash-1/Achaete-scute (1:500, Abcam, ab74065), rabbit anti-RFP (1:2000, Rockland, 600-401-379), mouse anti-iNOS (1:200, BD Bioscience, 610329), rabbit anti-Gas6 (1:25, R&D Systems, AF986), rat anti-Ki67 (1:200, Invitrogen, 14-5698-82), goat anti-ApoE (1:50, Invitrogen, PA1-26902), rabbit anti-ApoE (1:50, Abcam, ab183597), rat anti-CD45 (1:100, BD Pharmingen, 553079), mouse anti-EGFR (1:100, Merck Millipore, 05-1047), rabbit anti-Lrp8 (1:50, ThermoFisher, bs-6651R), rabbit anti-NeuN (1:500, Abcam, ab177487), mouse anti-Olig2 (1:200, Merck Millipore, MABN50), rabbit anti-S100β (1:2000, Abcam, ab868). Secondary antibodies used included donkey antibodies to rabbit, rat, guinea pig, mouse, sheep, and goat conjugated with Alexa Fluor 488, 594, or 405 (1:200, Jackson ImmunoResearch Laboratories).

For FACS sorting: anti-CD133 (biotinylated, 1:300, eBioscience), anti-CD11b (Clone: M1/70, Brilliant Violett 605, 1:300, Biolegend), anti-CD45 (Clone: 30-F11, APC-Cy7, 1:200, Invitrogen), anti-Ly6C (Clone: AL-21, anti-Alexa Fluor 700, 1:200, BD Bioscience), anti-CD3e (Clone: eBio500A2, PE, 1:300, eBioscience), anti-CD19 (Clone: MB19-1, PE, 1.200, eBioscience), anti-Ly6G (Clone:1A8, PE, 1:200, BD Bioscience), Fc Block (Clone: 2.4G2, 1:250, BD Bioscience), Live/Dead (DAPI, 1:10000).

Validation

All antibodies used in the study were from commercial sources and were validated by the vendors and previous studies performed by our laboratory or by others.

Animals and other research organisms

Policy information about studies involving animals; ARRIVE guidelines recommended for reporting animal research, and Sex and Gender in Research

Laboratory animals

C57BL/6N mice (Charles River) and transgenic mice (C57BL/6N background) were used. For the analysis of SVZ NSPCs, Nestin-CreERT2 mice15 were crossed with R26-yfp mice46, resulting in Nestin-CreERT2:R26-yfp mice. For the analysis of microglia, Cx3cr1-CreERT2 mice47 were crossed with R26-tdTomato mice to generate Cx3cr1-CreERT2:R26-tdTomato mice. To visualize microglia and monocytes, Cx3cr1-EGFP48, HexB-tdTomato49 and Ccr2-RFP50 transgenic mouse lines were used. For in vitro acute slice imaging, Nestin-CreERT2:R26-tdTomato mice were crossed with Cx3cr1-EGFP mice, resulting in Nestin-CreERT2-R26-tdTomato:Cx3cr1-EGFP mice.

Wild animals

This study did not involve wild animals

Reporting on sex

Female and male mice were used in this study.

Field-collected samples

This study did not involve field-collected samples

Ethics oversight

Adult mice of both genders were used. Animals were housed under Institutional Animal Care and Use Committee guidelines in a temperature and humidity-controlled facility with a 12 h light–12 h dark cycle and ad libitum feeding. All animal experiments were approved by the Federal Ministry for Nature, Environment, and Consumer Protection of the state of Baden-Württemberg (G16/110, G21/56) and were performed in accordance with the respective national, federal, and institutional regulations.

Note that full information on the approval of the study protocol must also be provided in the manuscript.

Plants

Seed stocks	NA
Novel plant genotypes	NA
Authentication	NA

Flow Cytometry

Plots

Confirm that:

- [x] The axis labels state the marker and fluorochrome used (e.g. CD4-FITC).
- [x] The axis scales are clearly visible. Include numbers along axes only for bottom left plot of group (a 'group' is an analysis of identical markers).
- [x] All plots are contour plots with outliers or pseudocolor plots.
- [x] A numerical value for number of cells or percentage (with statistics) is provided.

Methodology

Sample preparation	Sample preparation for FACS sorting were described in detail under the Methods section of this manuscript.
Instrument	Gallios FACS (Beckman Coulter), MoFlo Astrios cell sorter (Beckman Coulter)
Software	Gallios FACS (Beckman Coulter), MoFlo Astrios cell sorter and the Gallios/Navios analysers (Beckman Coulter)
Cell population abundance	Small debris was removed with the preliminary FSC/SSC gate. Single, living cells were obtained by doublet exclusion and exclusion of dead cells with live/dead staining with DAPI. For cell sorting, 1152 cells (containing microglia and NSPCs) were collected and pooled from each control and PT group and subsequent for scRNA sequencing.
Gating strategy	For microglia, cells were gated on CD11b+CD45+low and for NSPC, cells were gated on YFP+ subpopulation<br>Positive and negative gates were set using unstained and fluorescence minus one (FMO) background intensity controls. Fluorophores were chosen to minimize spectral overlap.

- [x] Tick this box to confirm that a figure exemplifying the gating strategy is provided in the Supplementary Information.
